# Numerical Study of an Efficient Solar Absorber Consisting of Metal Nanoparticles

**DOI:** 10.1186/s11671-017-2363-7

**Published:** 2017-11-22

**Authors:** Chang Liu, De Zhang, Yumin Liu, Dong Wu, Lei Chen, Rui Ma, Zhongyuan Yu, Li Yu, Han Ye

**Affiliations:** 1 State Key Laboratory of Information Photonics and Optical Communications, Beijing University of Post and Telecommunications, Beijing, 100876 China; 2 Information Science Academy of China Electronics Technology Group Corporation, Beijing, 100876 China; 3 School of Science, Beijing University of Post and Telecommunications, Beijing, 100876 China

**Keywords:** (350.6050) Solar energy, (300.1030) Absorption, (240.6680) Surface plasmons, (310.6628) Subwavelength structures, nanostructures, (160.3918) Metamaterials

## Abstract

We propose and theoretically investigate an efficient solar light absorber based on a multilayer structure consisting of tungsten nanoparticle layers and SiO_2_ layers. According to our calculation, average absorbance over 94% is achieved in the wavelength range between 400 and 2500 nm for the proposed absorber. The excellent performance of the absorber can be attributed to the localized surface plasmon resonance as well as the Fabry-Perot resonance among the metal-dielectric-metal layers. We compare the absorbing efficiency of tungsten nanosphere absorber with absorbers consisting of the other metal nanoparticles and conclude that iron can be an alternative material for tungsten in solar energy systems for its excellent absorbing performance and the similar optical properties as tungsten. Besides, a flat multilayer absorber is designed for comparison, and it is also proved to have a good absorbing performance for solar light.

## Background

Solar energy systems have drawn more and more attentions in recent decades due to the excessive consumption of traditional energy sources and seriously deteriorating environment situation. In solar energy systems, solar energy can be converted to electricity or thermal energy for different usages with minor pollution to the environment. However, the present solar energy systems, like thermophotovoltaic (TPV) systems, solar steam generation systems, solar water heating systems, are inefficient in energy conversion, and efficiency approaching 20% in appropriate optical condition has been theoretically predicted in TPV systems [1], which is still far away from being widely produced. Many high-efficient solar absorbers are developed to improve the energy conversion efficiency in kinds of solar energy systems. Surface plasmons polaritons (SPP), localized surface plasmons (LSP), and magnetic resonances are often utilized to realize near-perfect absorption in those absorbers. As solar light has a wide range of spectrum (from 200 to 3000 nm), it requires a broad enough absorbing spectrum for absorbers to effectively convert light. However, single resonance mode excited in many absorbers usually cannot result in broadband light absorption. The common solution to solve this problem is to design absorbers with multiple resonance modes. For example, multilayer systems, like flat metal-dielectric-metal (MDM) structures [2, 3], MDM pyramid multilayer structures [4, 5], or MDM with kinds of gratings structures [6], can often have broadband absorption resulting from multi-resonances excited between metal-dielectric layers as long as the number of layers is enough. Other structures, like arrays of minor absorbing structures [7, 8], or structures with gradient changing in their sizes [8], can support different resonance modes and also result in broadband absorption. Most of these designs require quite difficult fabricating processes, and the absorbing efficiency are very critical to the fabricated structure and the surrounding environment, which strongly inhabits their applications.

Besides, the materials of absorbers should be cheap enough, which can provide the possibility of a wide production. However, many reported absorbers use noble metals in their structure. Near-perfect absorption can often be achieved in these absorbers within the range of visible light, but their absorbing performance out of this region is terrible [9–13]. As there is more than 40% energy of solar light out of the visible light spectrum, these absorbers usually may be inefficient in solar energy systems. Besides, the melting points of noble metals like gold and silver are around 1000 °C, and they can be easily melt when applied in a high-temperature solar energy system, which seriously influences the stability and efficiency of a solar energy system. Therefore, the common metallic material used in solar energy system is tungsten. Compared with other metals, tungsten absorbers often have relative high melting point, have stable chemical properties, and show excellent performance in absorbing broadband solar light [14]. These advantages make tungsten an indispensable role in solar energy system.

In this paper, we propose a broadband solar light absorber based on the design of nanoparticle-dielectric multilayers and the application of tungsten and iron in the structure. The paper is arranged as follows. First, we will introduce the 3-D absorber and show the simulation results. Then, we will illustrate the absorbing mechanism of the absorber and compare this structure with the flat MDM structure to get a deeper insight. Further, there will be a discussion between iron nanoparticle absorber and tungsten nanoparticle absorber for their performance when applied in this structure.

## Methods

**Fig. 1:**
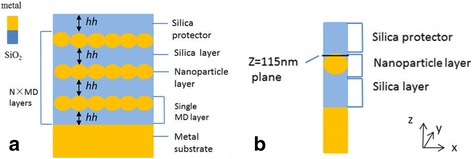
**a** Basic structure of metal nanoparticle-dielectric absorber (NPA). All of the dielectric layers have a thickness of hh (100 nm). The diameter dd of metal nanoparticles is 20 nm. **b** One unit cell of the single-MD-layer NPA structure. Period *P* = dd = 20 nm

The basic structure of the metal nanoparticle absorber (NPA) is depicted in Fig. 1a. The absorber is composed of multiple metal nanoparticle-dielectric (MD) layers. The metal nanoparticle layer consists of closely arranged nanoparticles of square array in cubic lattice embedded in SiO_2_ layer. The diameter of the nanoparticles is 20 nm, and there is no gap between the neighboring nanoparticles. The dielectric layer in the most top of the structure is used for protecting the metal particles from being oxidized. A unit cell of single-layer NPA is plotted in Fig. 1b. The top dielectric layer is for protecting the metal from being oxidized and has the same thickness as the lower dielectric layer. Thus, the metal particle is embedded in the middle of the whole dielectric layer. Tungsten is chosen to be the metallic part of the structure due to its excellent performance in TPV system [14], and we chose silica to be the dielectric part of the absorber for its relative low refractive index. Developing modern nanofabrication techniques, such as electron-beam lithography [15], focused ion beam milling [16], magnetron sputtering method [17], or self-assembly of colloids [18], makes it possible to produce nanoparticle layer structures proposed in this paper [19, 18, 20–22].

As for simulation, we use 3-D finite-difference time-domain (FDTD) method. The corresponding software is Lumerical FDTD. The refractive indexes of dielectric (SiO_2_) and metal (tungsten) are both adopted from the experiment data [23, 24]. As the metal nanoparticle layers consist of infinite continuous nanoparticles, we choose one metal nanoparticle cell as the simulation model. We plot a unit cell of the periodical single-layer NPA structure in Fig. 1b. A normally incident TM light is incident along the negative *y* direction with the polarization along the *x* direction. Therefore, the simulation period *P* is the same as the diameter of the metal nanoparticle (20 nm). The minimum mesh size is set as 0.1 nm. Periodical boundary condition is adopted for single unit cell in Fig. 1b. Perfect match layers (PML) are adopted at the bottom and top of the structure. The absorbance is calculated as *A* = 1 − *R* − *T*, where *R* is the reflection and *T* is the transmission. The thickness of the metal substrate is set as 300 nm, which is much larger than its typical skin depth to avoid transmitting light. Thus, there is nearly no transmittance in the overall frequency range, and the absorbance of the absorber can be calculated as *A* = 1 − *R*.

## Results and Discussion

**Fig. 2:**
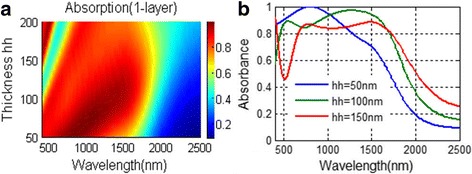
**a**, **b** Absorbing performance for one-layer NPA varying with dielectric thickness hh

For one-layer NPA, the absorbing performance is depicted in Fig. 2 varying with the dielectric layer thickness hh. In Fig. 2, two distinguished regimes are observed, namely the thin-dielectric-layer regime (hh < 100 nm) and thick-dielectric-layer regime (hh > 100 nm). At the thin-dielectric-layer regime, the well-absorbing band is broadened with the increase of the thickness hh. However, at the thick-dielectric-layer regime, there is an absorbing dip appearing at a shorter wavelength range and the well-absorbing area shrinks as the dielectric layer is getting thicker. We choose hh = 100 nm in our following study due to relative well-absorbing performance over the operating band and also due to no obvious absorbing dip appearing in the visible region.

**Fig. 3:**
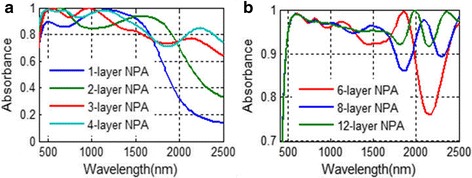
**a**, **b** Absorbance of the NPA structure with multiple layers applied. *N*-layer NPA means that NPA with *N* MD pairs

When there is only one MD layer in the structure, absorbance over 80% is achieved for the wavelength range from 400 to over 1600 nm, which already exceeds many solar absorbers reported. With more MD layers applied, the absorbing performance of the absorber can be further improved. We plot the absorbing performance of NPA with different numbers of MD layers in Fig. 3. With more MD pairs applied to the NPA structure, the absorption in longer operating wavelength increases greatly. With four MD layers applied, the absorbance of the corresponding absorber can almost exceed 80% for the wavelength range from 400 to 2500 nm in which most of the solar light spectrum is included. With eight MD layers applied to the NPA, an absorbance over 90% is obtained in most of the wavelength range from 400 to 2500 nm. With 12 MD pairs applied to the NPA, the absorption exceeds 90% in the whole operating wavelength.

**Fig. 4:**
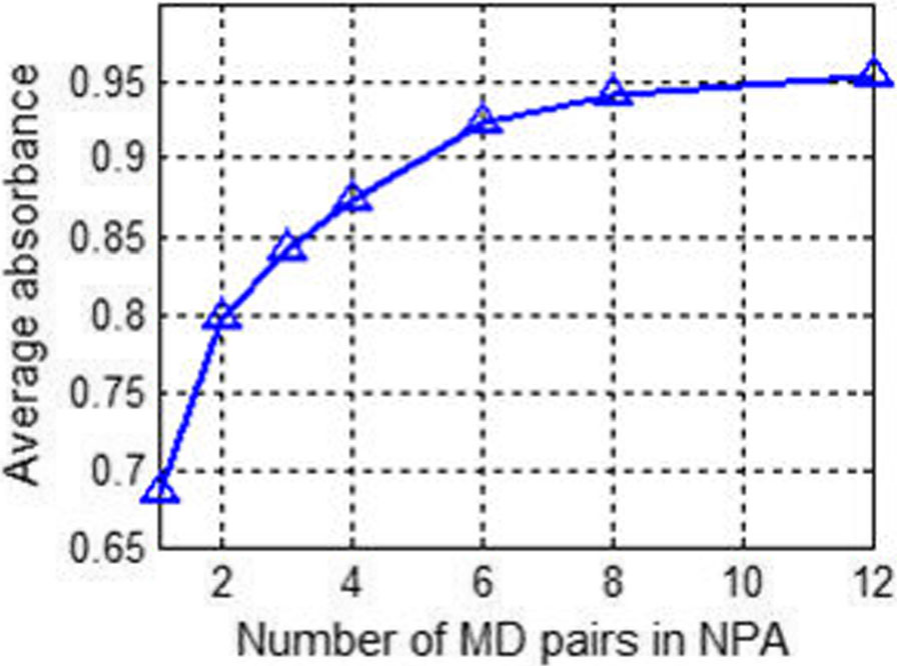
Average absorbance as a function of the number of MD layers

To further illustrate the relationship between the absorbing performance of NPA absorber and the number of the MD pairs in the NPA structure, we calculate the average absorbance of NPA absorbers varying with different numbers of MD pairs. The average absorption can be calculated as

$$ \overline{A}={\int}_{\lambda_2}^{\lambda_1}A\left(\lambda \right) d\lambda /\left({\lambda}_1-{\lambda}_2\right) $$

where *λ*_1_ and *λ*_2_ is 2500 and 400 nm, respectively, at our case. The relationship between the number of MD layers and the average absorption is depicted in Fig. 4. With the increment of MD pairs, the average absorption rises from 68.5% (single MD layer) to 95.4% (12 MD layers). When the number of MD pairs is more than 8, the growth of the average absorption seems to reach its instinctive limit and will be relatively slow. According to the calculation, the average absorbance of NPA with more than five MD layers reaches up to 90% over the wavelength range of 400 to 2500 nm. This absorber exceeds many of previously reported absorbers in both absorption efficiency and perfect absorption bandwidth.

**Fig. 5:**
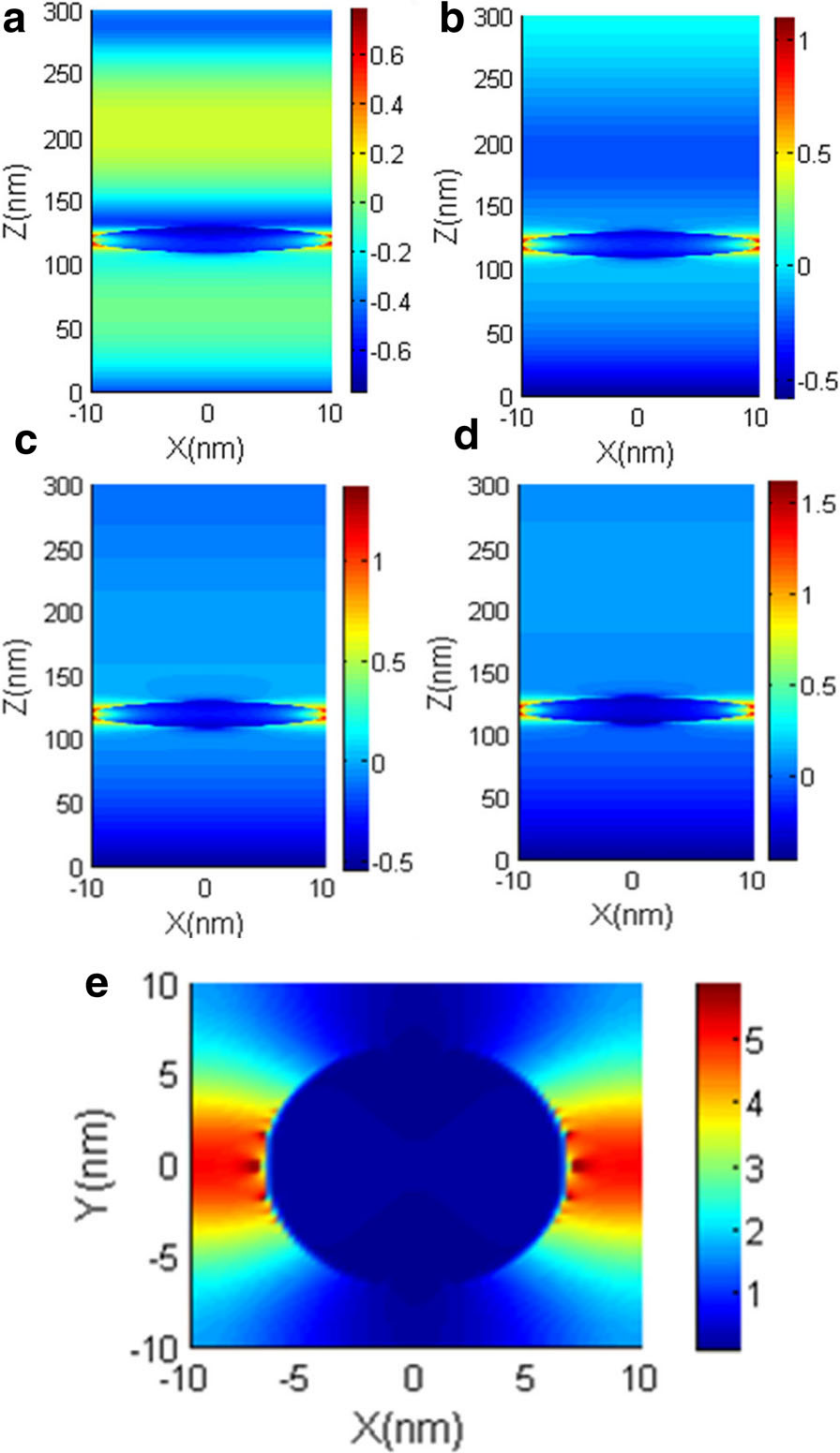
Electric field magnitude distribution (log_10_|*E*/*E*_0_|) of single-MD-layer NPA: Electric field magnitude distribution in *y* = 0 plane at wavelength **a** 440 nm, **b** 750 nm, **c** 1150 nm, and **d** 1580 nm; **e** electric field magnitude distribution (|*E*/*E*_0_|)in *z* = 115 nm plane at wavelength 905 nm

As we mentioned before, the NPA structure can realize high absorption even with only one MD pair. To understand the physical mechanisms responsible for the high absorption of the single-layer NPA structure, we plot its spatial distribution of electric field in Fig. 5. Figure 5a is the electric-field magnitude distribution of the single-layer NPA structure in plane *y* = 0. With incident light polarized along *x* direction, the electric filed is enhanced and confined around the nanoparticles. Such a field profile suggests that the absorption can be ascribed to the localized surface plasmon resonance (LSPR) [25]. To better show that, we plot the cross-sectional electric-field magnitude distribution of the particle in the *z* = 115 nm plane (marked in Fig. 1b) in Fig. 5e. Clearly, electric field enhancement appears at the both sides of the metal particles along the polarization direction of incident light. Due to the nanoparticles are closely arranged, the LSPR around the particles coupling with neighboring LSPR together result in high absorption of the NPA structure. The coupling of neighboring LSPR consumes light and results in the high absorption of the NPA structure.

**Fig. 6:**
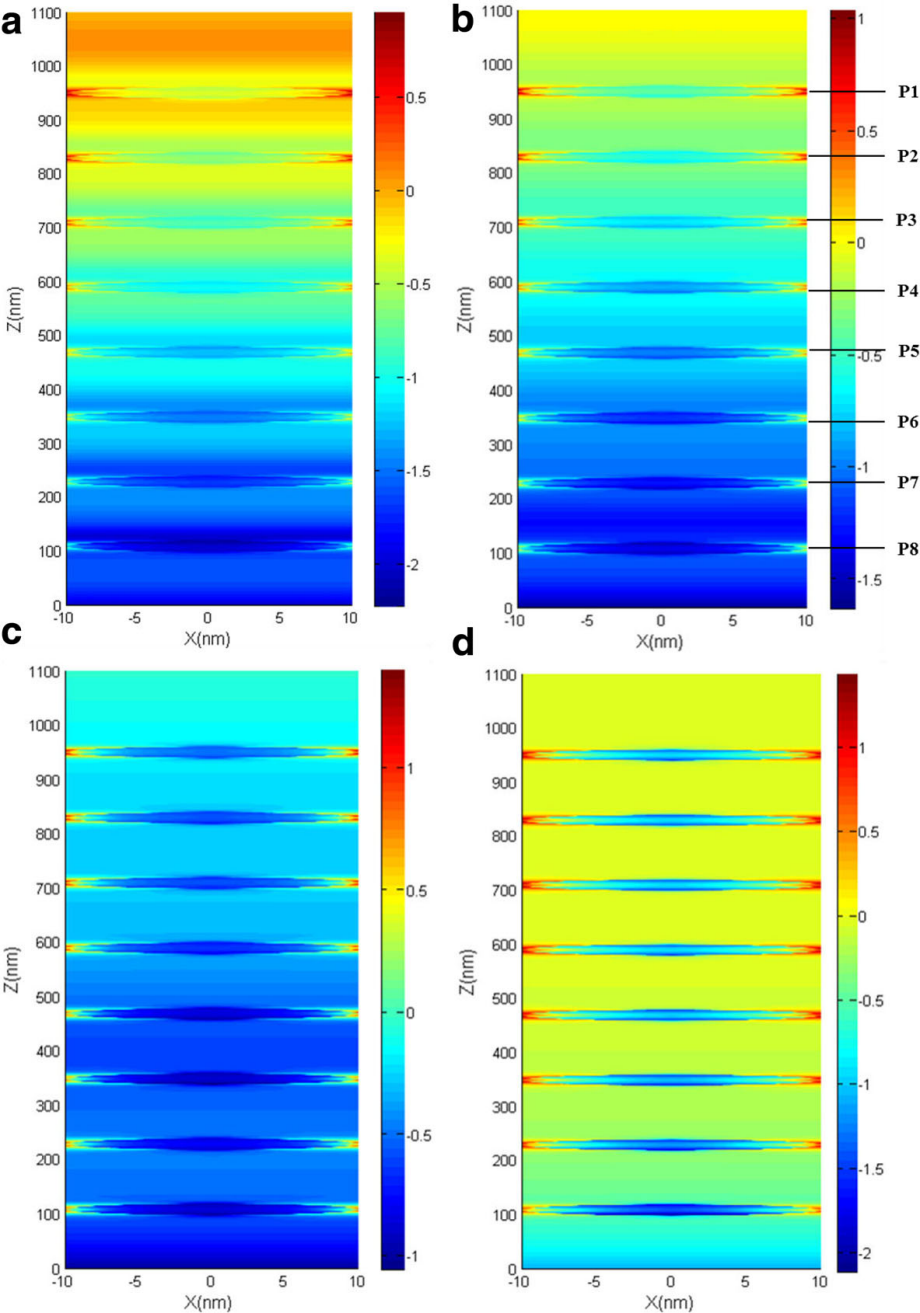
Electric magnitude distribution (log_10_|*E*/*E*_0_|) of the eight-MD-pair NPA structure in the *y* = 0 plane at **a** 441 nm, **b** 638 nm, **c** 1580 nm, and **d** 2500 nm. p1–p8 represent the eight particles in the one unit cell of the eight-MD-pair NPA structure

Compared with the single-MD-pair NPA, the absorbing performance is greatly improved in the longer wavelength range for the NPA structure with multiple MD pairs. To illustrate this phenomenon, we plot the spatial electric distribution of the eight-MD-pair NPA structure at Fig. 6. For light of different wavelengths, the field magnitude distributions are different. For shorter-wavelength light (Fig. 6a, b), it is mainly absorbed by the upper MD layers. The electric filed magnitude and field confinement around the nanoparticle in lower layers of the structure is weak. While for longer wavelength (Fig. 6c, d), the electric field confinement exists obviously in all of the MD layers and LSPR appearing strongly around not only the upper particle layers, but also the lower particle layers. This means that for the multiple-MD-pair NPA structure, the lower MD layers do not participate well into absorbing shorter-wavelength incident light. Instead, the longer-wavelength incident light can be well absorbed and transformed into LSPR in the lower MD layers. Thus, adding MD pairs to the NPA structure will greatly improve the absorbing performance of the NPA structure for longer-wavelength light, which corresponded well to the absorbing curve in Fig. 3a. Also, this explains the reason why the absorbing curves for different MD pairs in the NPA structure in Fig. 3b increase apparently in the longer wavelength range but merge together in shorter wavelength with the increment of MD pairs.

**Fig. 7:**
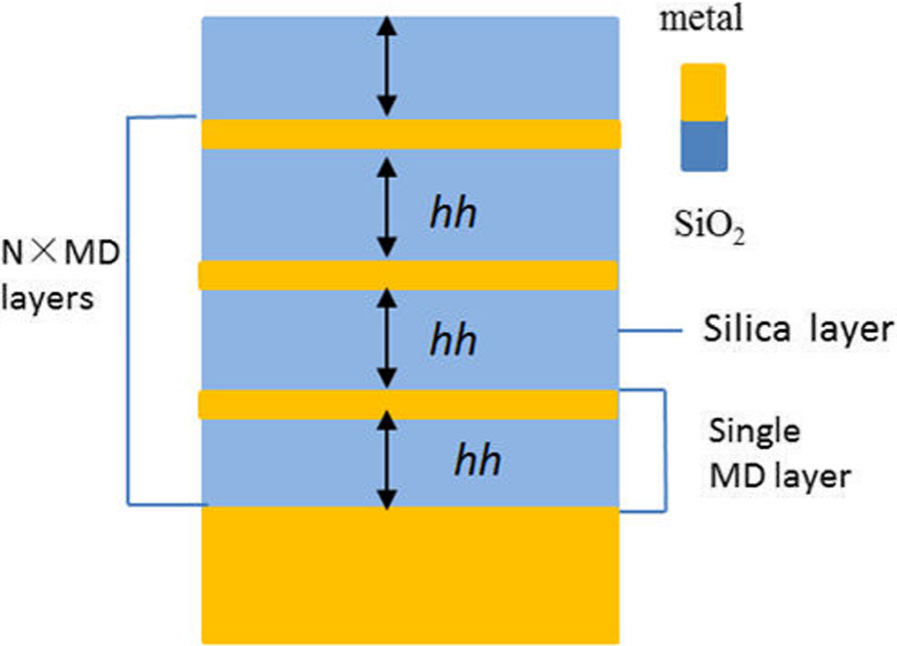
Diagram of flat metal-dielectric multilayer absorber (FMA)

**Fig. 8:**
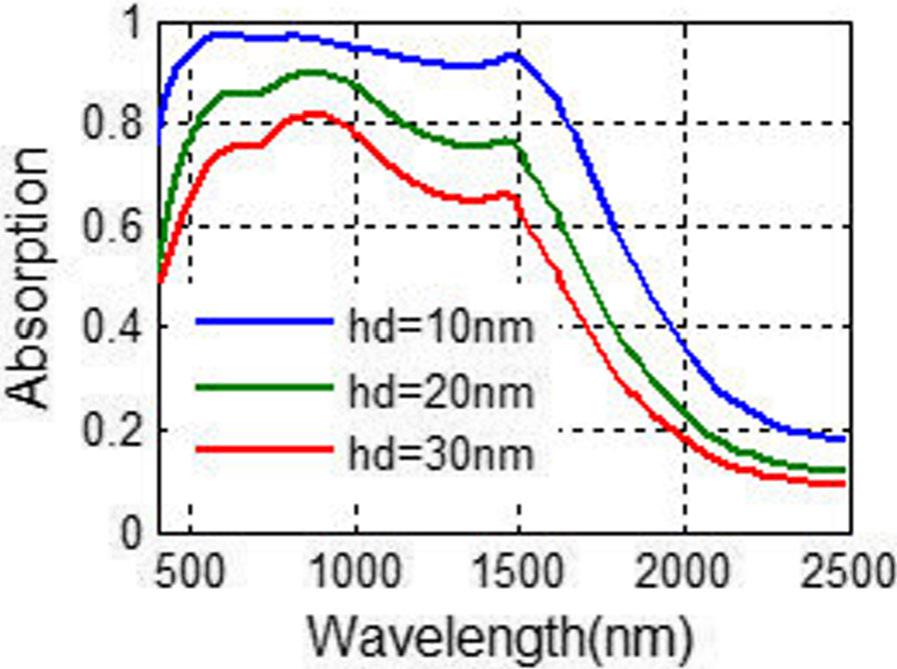
Absorbing spectra of the eight-MD-pair FMA varying with the metal thickness hd. The dielectric layer thickness hh is set as 100 nm

To get a deeper insight of the NPA structure, we calculate the absorbing performance of a similar absorber—FMA (flat MDM absorber, plotted in Fig. 7). The absorbing spectra at different metal layer thicknesses hd has been plotted in Fig. 8. The layer thickness of SiO_2_ is set as 100 nm, which is the same as the NPA structure. With thicker metal layers, the absorbance of the FMA structure turns to decrease. The absorbance over 90% is achieved for the wavelength range from 400 to 1500 nm when hd = 10 nm. However, when the metal layer thickness hd is set as 20 nm, which is the same as the metal layer thickness of the NPA structure, the absorbing efficiency of FMA drops obviously. This can be easily understood, because when the metal layers are getting thicker, the reflectance of the structure is more obvious and the absorbance reduces as a result. The selective absorption of FMA is better than NPA. When the wavelength is over 2500 nm, the absorption is below 20%. Although there are lots of MDM absorbers proposed for solar light absorbing [26, 27–32], the absorbing performance of our FMA exceed many other MDM absorbers. The absorption efficiency of FMA is high, and the absorbing bandwidth is quite broad. Another advantage of MDM is the absorbing selectivity of FMA. When the wavelength is over 2500 nm, the absorption is below 20%, which makes it able to be applied in the selective solar energy systems, like TPV systems. Besides, the thickness of the metal layers in FMA is 10 nm, which is thicker than the MDM absorber in refs. [31, 32] and makes it more easy to be fabricated. These advantages are all due to the application of tungsten in the FMA structure instead of noble metals which are commonly used in MDM absorbers.

**Fig. 9:**
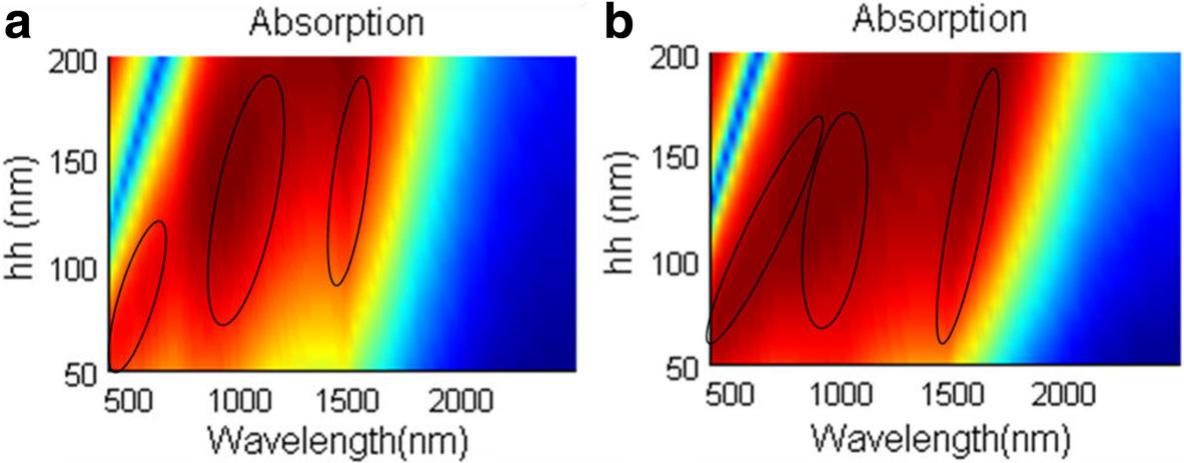
Absorbing spectra of the three-layer FMA as **a** hd = 20 nm and **b** hd = 10 nm varying with dielectric thickness hh. Black circles mark resonance peaks

For the MDM absorbers, their absorbing abilities for light are often based on the Fabry-Perot resonance [2, 6, 33]. When adding more MD pairs to the structure, there is an extra absorbing peak appearing at the absorbing spectrum for FMA due to Fabry-Perot resonance. To better show this, we plot three-layer FMA as an example. Figure 9 plots the absorbing performance of three-layer FMA varying with dielectric thickness hh. For both Fig. 9a and Fig. 9b, there are three absorbing peaks appearing in the spectrum, which results from Fabry-Perot resonance [2, 6]. The resonance wavelength of Fabry-Perot resonance increases with the cavity thickness [2, 6]. Herein, the absorption band broadens to longer wavelength range with the increment of dielectric layer thickness hh, and the absorption band has a redshift in Fig. 9.

**Fig. 10:**
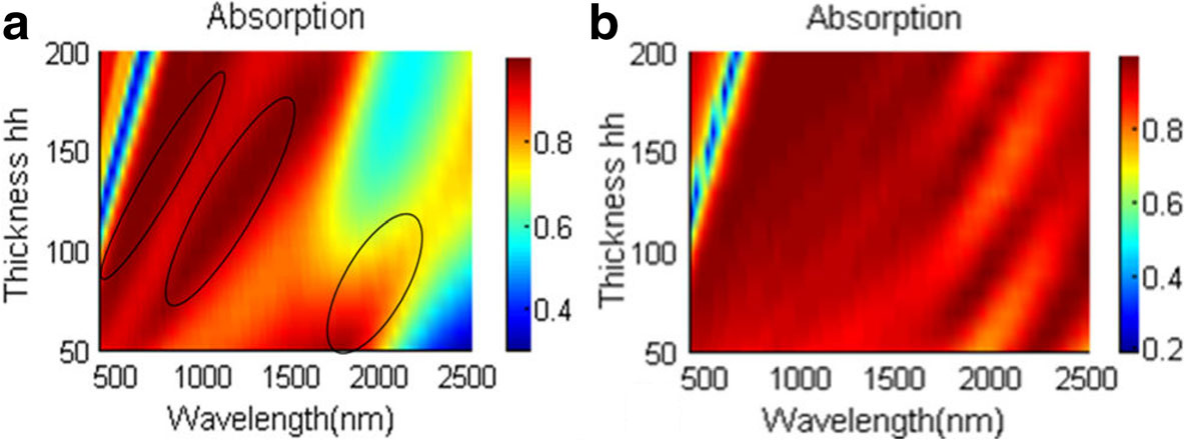
The absorbing spectra varying with silica layer thickness hh in **a** the three-layer NPA structure and **b** the eight-layer NPA structure

This also happens to the NPA structure. For absorbing spectrum in Fig. 2a, the absorbing peak appearing around 1000 nm should be the result of Febry-Perot resonance. When there are three MD pairs in NPA, there will also be three absorbing peaks in the absorbing spectrum (show in Fig. 10) as the absorbing spectrum of three-layer FMA in Fig. 9. However, when eight MD pairs are applied to NPA, the absorbing peaks merge together; there are only several absorbing peaks that can be observed in longer wavelength. When increasing the thickness of the dielectric layer in Fig. 10, the absorbing spectrum redshifts. Due to the similarities of the absorbing spectrum of the three-layer FMA and NPA, we can infer that the excellent absorbing performance of NPA should also result from the Fabry-Perot resonance. Therefore, there are both LSPR and Fabry-Perot resonance in NPA. The excellent absorbing performance should be the result of the existence of LSPR and Fabry-Perot resonance.

**Fig. 11:**
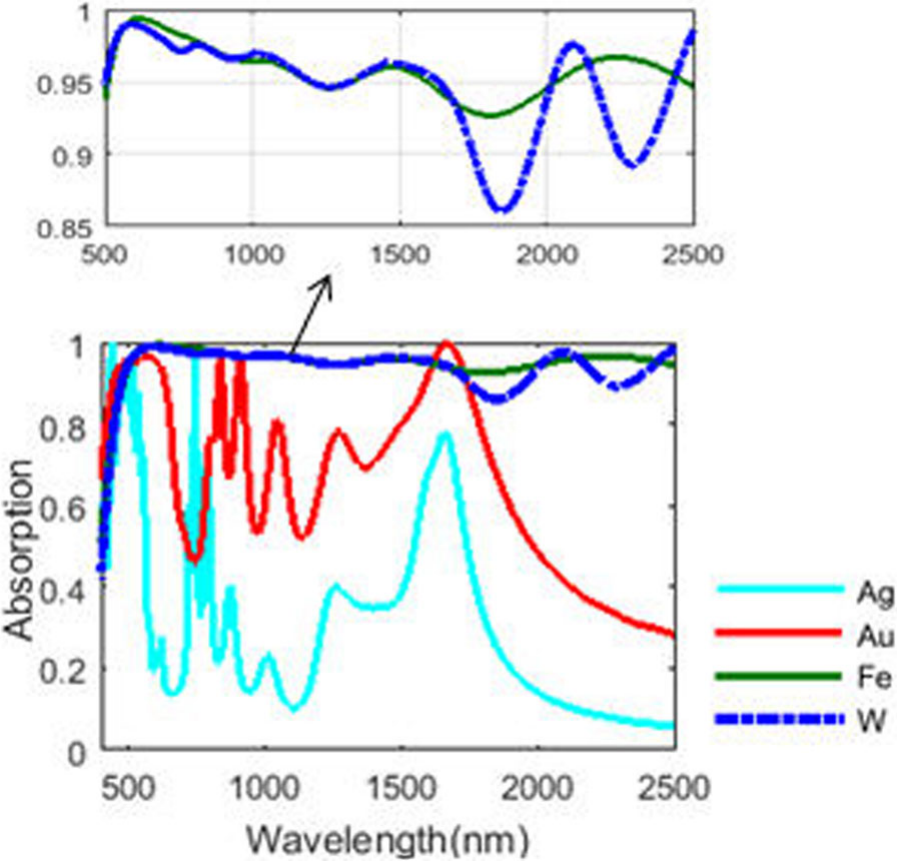
Absorbance of the eight-layer NPA structures with different metals applied

The metal we choose for this absorber is tungsten. In our previous work [34], we have shown that iron can be an excellent candidate to be applied in the solar light absorbers. As depicted in Fig. 11, we compare the absorbing performance of tungsten nanoparticle structure with the performance of absorbers consisting of other metal nanoparticles under the same structure. An absorbing efficiency over 92% for the wavelength range from 400 to 2500 nm is achieved for iron absorber. The well-absorbing bandwidth of iron absorber (about 2.1 μm) exceeds the bandwidth of tungsten absorber (about 1.8 μm). The absorbing efficiency of the golden absorber and silver absorber merely reach 90% within narrow wavelength ranges. Their absorbing performances are much worse than the tungsten and iron absorbers under this structure. This result corresponds well to our former work [34], which also shows that iron absorber often has better absorbing performance over noble metals due to the well-matching condition between the impendence of iron absorber and the impendence of free space. Noble metals are well known for their excellent absorbing performance of visible light in the field of solar light absorption. However, they are usually not used in TPV system as an absorber or emitter, because they are unable to absorb well the light out of the visible light range. Besides, their melting points are relatively low (around 1000 °C), which seriously hinders their applications in solar energy systems.

**Fig. 12:**
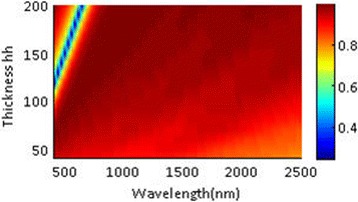
Absorbing spectra varying with layer thickness hh in the eight-layer Fe-NPA structure

**Fig. 13:**
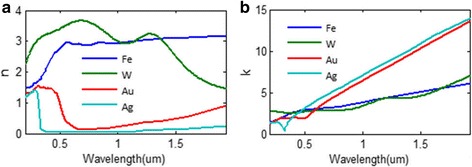
Comparing of the **a** real part of refractive index and **b** the imaginary part of refractive index of commonly used metals

Like tungsten NPA structure, the absorbing spectrum of iron NPA structure also has a redshift with the increase of silica layer thickness hh (plotted in Fig. 12). The absorbing efficiency is almost beyond 90% for the entire operating waveband apart from an absorbing dip of a 100-nm wavelength range appearing when the layer thickness hh is over 100 nm. Compared with Fig. 7, the overall absorbing performance of the iron NPA structure exceeds that of the tungsten NPA structure. The average absorption of iron nanoparticles (94.88%) and tungsten nanoparticles (94.09%) exceed that of gold (64%) and silver (28.4%) nanoparticles. The excellent absorbing performance makes iron a promising alternative material for tungsten in solar energy system. Besides, iron is more cost-effective than tungsten. Its melting point is around 1500 °C and is higher than that of noble metal. For tungsten, the chemical stability is one of the crucial properties in solar systems. Alloy of iron and tungsten may have the advantages of the two metals. We further compare their reflective indexes in Fig. 13. Data of gold and silver are adopted from reference [35]. It shows that the optical properties of tungsten and iron are very similar especially for the imaginary part of their reflective indexes, which results in their similar absorbing performances in the NPA structure.

**Fig. 14:**
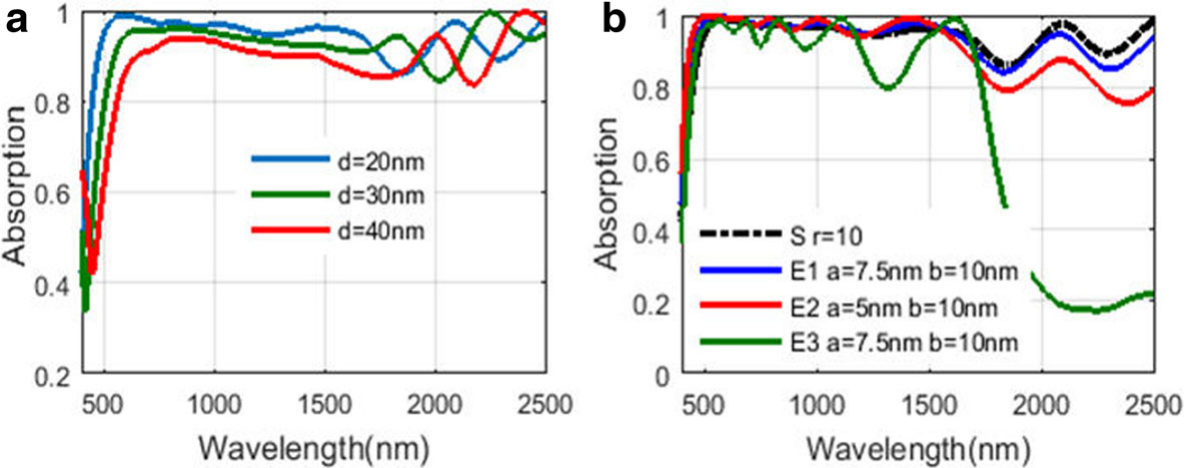
**a** Absorbing spectrum of the NPA structure varying with nanoparticle size. **b** Absorbing spectrum of the NPA structure for nanoparticles with different shapes. *S* sphere, *E* ellipsoid, *a* the major axis semidiameter of the ellipsoid, *b* is the minor axis semidiameter of the ellipsoid. For E1 and E2, the electric filed is along the major-axis direction. For E3, the electric field is along the minor-axis direction

For the NPA structure, the fabrication of such uniform small particles may be difficult. Therefore, well robustness is required for the proposed structure. We calculated the absorbing performance of structures consisting of different shapes and sizes in Fig. 14a, b. For different sizes of nanoparticles, the absorption of the structure remains over 90% at almost of the operating wavelength. When we change the spherical nanoparticles into ellipsoid nanoparticles in the NPA structure, the absorption decreases (shown in Fig. 4b). For E1 and E2 conditions in which the electric field is along the major axis of the ellipsoid particles, the absorption drops mainly in the wavelength range over 1700 nm and the absorption in the shorter wavelength where most of the solar energy is distributed almost remains the same. The average absorptions in these two cases are over 90%. When the electric field is along the minor axis of the ellipsoid particles, the absorption changes dramatically. Therefore, the direction of the major axis of the ellipsoid-shape nanoparticle should be kept to agree with the direction of the electric field while fabrication.

**Fig. 15:**
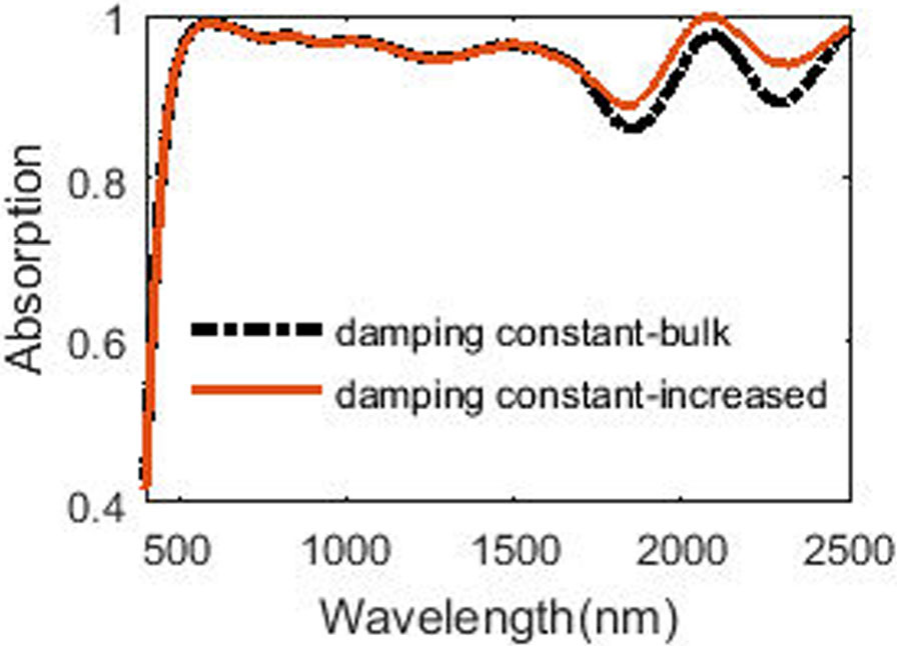
Absorption using different damping constant of tungsten

Besides, the damping constant of tungsten nanoparticle is often larger than the bulk tungsten due to surface scattering and grain boundary effects. According to the data in reference [36], we recalculate the absorption of the structure using the increased damping constant of tungsten. The result is plotted in Fig. 15. When the damping constant of tungsten increases, the absorption in the shorter wavelength (from 400 to 1700 nm) remains almost unchanged, while the absorption in the longer wavelength (from 1700 to 2500 nm) increases. This can be attributed to that when the damping constant of tungsten in the infrared region increases, the imaginary part of its permittivity in the infrared region will increase [36] and result in the increment of the absorption. The change of the permittivity of tungsten is more obvious in longer wavelength than shorter wavelength. Therefore, the absorption calculated with the increased damping constant in longer wavelength changes a little while it in the shorter wavelength nearly remains unchanged.

So far, we have discussed the NPA structure and FMA structure and their absorbing performances and absorbing mechanism and the metals that can be applied in them to reach high absorption. However, the applications of these absorbers may be different. In TPV system, the well selective absorbing characteristics are often required to reduce thermal emission from the solar absorber. So, multilayer NPA structures whose absorbing performances are plotted in Fig. 3b are not suitable to be used in TPV system due to high thermal emission over 2500 nm. However, the NPA structure with a few MD layers (absorbing spectrum plotted in Fig. 3a) and FMA structure (absorbing spectrum plotted in Fig. 9) can be used in the TPV system due to low thermal emission over 2500 nm. For multilayer NPA structures, they could be useful in other solar energy systems in which well selective absorbing performances are not required, like solar steam generation [37], waste water treating systems, and water heating systems.

## Conclusions

In summary, we have proposed a highly efficient broadband absorber consisting of tungsten nanoparticle layers and SiO_2_ layers on the top of a metal substrate. With eight MD layers applied, the absorber can have an absorbance over 90% for most of the wavelength range from 400 to 2500 nm. The absorbing efficiency of this absorber exceeds the absorbing efficiency of many other solar light absorbers, which provide much possibility for the absorber to be applied in solar energy systems like solar steam generation, solar water heating, and waste water treating systems. Also, we compare the NPA absorber with FMA and found that the excellent absorbing performance of NPA absorber results from LSPR and Fabry-Peort resonance. We further compare the absorbing performance of several common metal nanoparticle absorbers under the same structure parameters. Results show that iron can be a promising candidate material for tungsten in solar absorber. All these simulation results help to the design novel solar light absorbing cells in solar energy systems, and the absorbers we proposed are promising to be applied in the real applications.

## Abbreviations

FDTD: Finite-difference time-domain
FMA: Flat metal-dielectric multilayer absorber
LSP: Localized surface plasmon
NPA: Nanoparticle absorber
TPV: Thermo-photovoltaic
